# Low consistency refined ligno-cellulose microfibre: an MFC alternative for high bulk, tear and tensile mechanical pulp papers

**DOI:** 10.1007/s10570-019-02956-2

**Published:** 2020-01-14

**Authors:** Emilia S. Jahangir, James A. Olson

**Affiliations:** Pulp and Paper Centre, The University of British Columbia, 2385 East Mall, Vancouver, BC V6T 1Z4 Canada

**Keywords:** Thermomechanical pulp (TMP), Microfibrillated cellulose (MFC), Low consistency refining (LC), Tear index, Tensile energy absorption (TEA), Pulp reinforcement

## Abstract

Low consistency (LC) refining of (chemi-)thermomechanical pulp (TMP) provides an energy efficient alternative to high consistency refining for pulp property development. However, the benefit of LC refining is often limited by excessive fibre shortening, lower tear strength and a reduction of bulk caused by the refining process. In this study, microfibres produced by LC refining of TMP and kraft pulp fibres were investigated for their reinforcement potential in high freeness mechanical pulp. Primary pulp at 645 mL Canadian Standard Freeness was LC refined to different energy targets as a baseline for mechanical and optical property development. In contrast, the same primary pulp was reinforced with different microfibre types in ratios that yielded the same specific energies of the baseline LC refined pulp. The study revealed that at equivalent energies, the addition of TMP microfibres to the high freeness primary pulp displayed tensile development identical to the LC refined pulp, with significantly improved tear and bulk. The addition of kraft microfibre to primary pulp produced the highest tensile and tear strength but compromised light scattering. Additionally, all microfibre composites showed improved elongation, as opposed to no notable change in elongation with conventional LC refining. This investigation proposes an alternative, cost-effective approach for developing high bulk, high strength mechanical pulp by limiting the extent of second stage refining and using LC refined microfibres for pulp reinforcement. The high tear–high bulk open construction of the composite paper is likely to benefit boxboard and packaging applications.

## Introduction

Thermomechanical pulping (TMP) is an energy-intensive process that utilizes mechanical force to convert woodchips into high yield fibres. The traditional practice typically involves multiple stages of high consistency (HC) refining—a primary stage to separate fibres from the wood matrix and additional stage(s) of secondary refining to develop structural and surface properties of the fibres. In recent years, the secondary HC refining stages are increasingly being replaced by one or more stage(s) of low consistency (LC) refining due to the energy saving potential of the latter. Compared to HC refining, LC refining significantly reduces specific energy demand to develop pulp properties, such as tensile strength and freeness. Studies have reported a 20% or greater energy savings in mechanical pulp refining when second-stage HC was replaced by one or more stages of LC refining (Eriksen and Hammar 2007; Gorski et al. 2012; Sabourin 2007). The benefit of incorporating a third stage LC after two stages of HC in TMP refining has also been reported (Musselman et al. 1997).

As LC refiners are increasingly being installed in TMP mills, optimum refining conditions and process optimization of mechanical pulping system as a whole are frequently being explored in order to maximize energy savings in TMP pulp quality development (Elahimehr et al. 2013, 2015; Luukkonen et al. 2012; Rubiano Berna et al. 2019a, b). A number of studies have explored the effects of intensities in LC refining where energy is applied through either a small number of high-intensity impacts or a large number of low-intensity impacts. Compared to low-intensity, high-intensity LC refining is more desirable due to its relatively higher production rate and lower operating cost, however, higher intensity refining can be detrimental to pulp quality due to excessive fibre shortening, lower tear strength and reduction of bulk (Elahimehr et al. 2015; Hammar et al. 2010; Luukkonen et al. 2010; Miller et al. 2017; Olson et al. 2003; Welch 1999).

Another approach to reduce energy consumption in the TMP process while maintaining or improving pulp quality is to incorporate chemical treatments with mechanical pulping and refining. Studies showed that impregnating wood chips with certain chemicals can significantly improve tensile and tear strength of primary pulp during HC refining (Chang et al. 2016b), or during multi-stage LC refining of the primary pulp (Sun et al. 2016). This in turn, leads to reduced energy requirement to obtain pulp at a given tensile strength. Energy savings from chemical treatment of primary TMP prior to LC refining has also been reported in the literature (Chang et al. 2016a).

Mechanical pulp is often reinforced with expensive long-fibred chemical pulp to improve runnability and strength properties of the paper (Asikainen 2013; Hiltunen and Paulapuro 2011; Lehto et al. 2010a, b; Sjöberg and Höglund 2007). Studies led by Lehto et al. (2010a, b) demonstrated through laboratory and pilot experiments that both mechanical and chemi-mechanical pulp can be modified to reinforce paper, however, the higher fibre strength of chemical pulp makes it preferable for reinforcement, especially in terms of tear index and fracture energy. A more recent approach to improving strength properties of paper is nano and micro cellulose fibre reinforcement. Both microfibrillated cellulose (MFC) and cellulose nanocrystals (CNC) are widely explored cellulose derivatives that have shown exceptional reinforcement capabilities in various bio-composite systems, including paper products. The reinforcement capability of cellulose nanofibres or nanofibrillated cellulose in paper is well documented in the literature (Balea et al. 2019; Kajanto and Kosonen 2012; Sehaqui et al. 2013). Studies on MFC have shown that the addition of MFC produced from bleached and unbleached kraft pulp improves tensile strength, but reduces light scattering and brightness of kraft and TMP-based handsheets (Eriksen et al. 2008; Meyer et al. 2018). Several studies have focused on the production of MFC from TMP and other lignin-rich wood fibres. One such study, led by Spence et al. (2010), found that refining followed by high-pressure homogenization did not yield highly individualized MFC from TMP fibres. Tensile index improved for all MFC films produced from TMP and different lignin containing kraft furnish, compared to their original form; however, film prepared from TMP MFC had significantly lower tensile index than the rest. In a different study, Brodin and Eriksen (2015) explored the effects of fractionation and chemical treatment prior to homogenization in producing TMP-based lignocellulose micro-fibrils (LCMF). Tensile index and light scattering of low freeness TMP improved with the addition of all but one LCMF. Carboxymethylation followed by homogenization produced individualized LCMF from TMP fines and long fibre fractions and led to the highest tensile but lowest light scattering coefficient. Increase in tensile and opacity were also reported when TMP-based MFC was added to kraft handsheets (Meyer et al. 2018).

As MFC and its applications are emerging throughout the industry, from strength additives to packaging, pharmaceuticals to electronics, researchers are exploring economically viable methods to produce MFC. While some researchers have demonstrated energy savings in MFC production by incorporating enzymatic, chemical and mechanical pre-treatments with traditional homogenization and microfluidization techniques (Ankerfors 2015; Meyer et al. 2018; Spence et al. 2011), others have explored the prospect of manufacturing MFC through conventional refining of wood pulp. Researchers at the University of British Columbia’s Pulp and Paper Centre have successfully manufactured highly refined microfibres through mechanical disintegration of northern bleached softwood kraft (NBSK) pulp using a pilot-scale LC refiner (Khan et al. 2014). The resulting fibres, referred to as MFC, have significantly improved the tensile strength and tear resistance of sodium alginate-based films when added in small dosages. The strength-enhancing ability of this mechanically refined high energy fibres demonstrates the potential of LC refining in manufacturing an economically feasible MFC alternative as a strength additive for the pulp and paper industry.

In this study, we investigated the potential of mechanical and kraft pulp derived microfibres produced in a pilot-scale LC refiner as reinforcements for mechanical pulp-based paper. We explored pulp property development of a high freeness mechanical pulp reinforced with the different microfibre types and compared that with the property development of the same high freeness pulp when LC refined to different specific energy targets. Additionally, we compared the strength-enhancing abilities of mechanical pulp-derived lignin-rich microfibres with that of kraft pulp-derived cellulose microfibre in the high freeness mechanical pulp-based paper.

## Materials and methods

Experimental work was conducted at the Pulp and Paper Centre, the University of British Columbia in Vancouver, Canada, with the exception of microscopic imaging, which was done at the UBC Bioimaging Lab on campus. Microfibres were prepared by the application of high specific energy through multiple passes in a pilot-scale LC refiner, without any chemical pre-treatment or subsequent processing. Mechanical pulp used in this experiment was a high freeness, primary stage HC refined, never-dried TMP with spruce, pine and fir blend. Kraft microfibre (KMF) was prepared from dried NBSK pulp sheets.

### Pulp preparation through low consistency refining

**Fig. 1:**
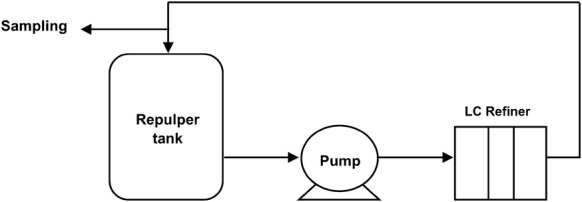
Schematic of Pulp and Paper Centre’s continuous recirculation flow loop

Refining trials were carried out using an AIKAWA 14” single-disc LC refiner with 16” overhung plates, and a 112 kW variable frequency drive motor. Trials utilized a small continuous recirculation flow loop equipped with a 250 L capacity repulper tank and a centrifugal pump (Fig. 1). Two sets of FINEBAR^®^ plates were used to facilitate different stages of refining. No-load power—defined as the refiner operational power—was determined at target refining conditions of 1200 RPM disc speed and 250 L/m flow rate, but at a wide 3.5 mm open plate gap, for each set of plates and pulp furnish. Net power was obtained by subtracting no-load power from the refiner gross power and used in subsequent calculations of specific refining energy (SRE) and refining intensity.

**Fig. 2:**
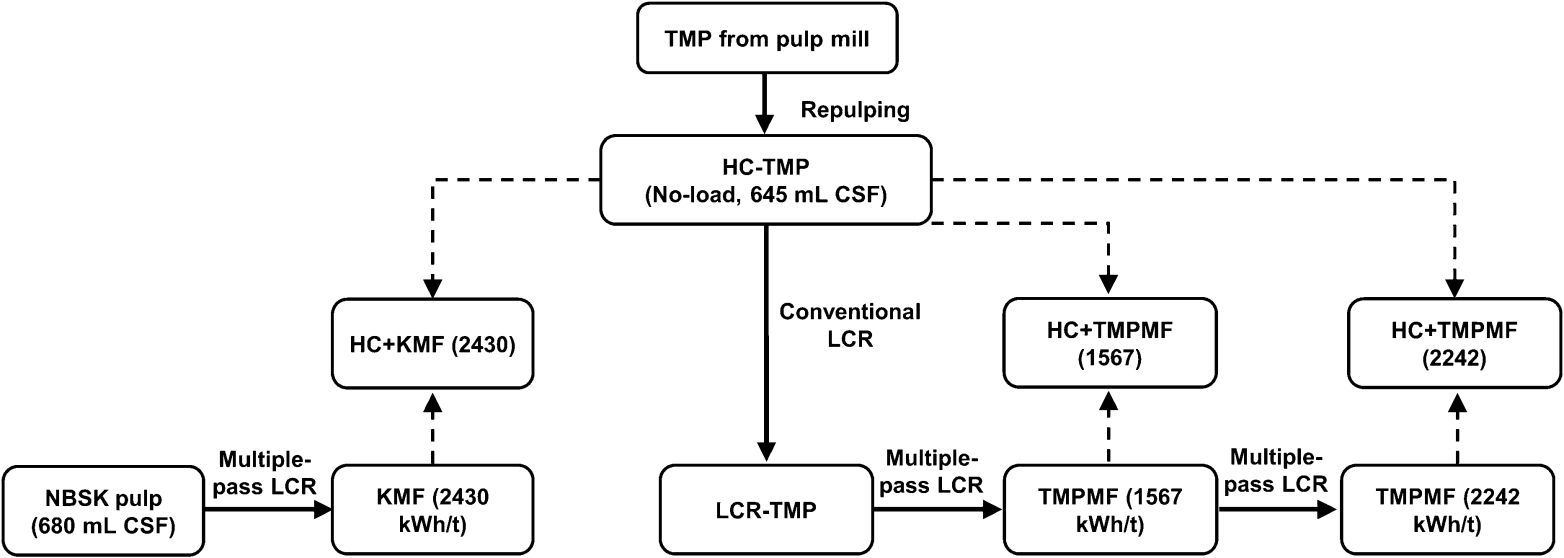
HC-TMP at different stages of pulp preparation; solid arrows indicate repulping and refining stages (LCR) whereas dotted arrows indicate the preparation of microfibre composites through a combination of HC-TMP and one of the three microfibre types—TMPMF (1567), TMPMF (2242) or KMF (2430)

The current study investigated pulp property development of a high freeness mechanical pulp through conventional LC refining as well as three different categories of microfibre reinforcement. First, the high freeness TMP from a commercial pulp mill was repulped in 45–50 °C tap water at about 3.5% consistency. To further facilitate the mixing and dispersion, pulp suspension was then recirculated through the LC flow loop at the no-load condition, ensuring no refining action was carried out. Once dispersed, no-load power was recorded, and a sample was collected and labelled as ‘HC-TMP’ for later use. The properties of HC-TMP are provided in Table 1. Since net power at the no-load condition is zero, by definition, the SRE of the pulp obtained at this stage was calculated to be 0 kWh/t, in the LC refining context. Energy imparted in the initial HC pulping and refining process is ignored in the course of this study. This zero-SRE HC-TMP, also referred to as the ‘primary pulp’, is the base material for all four categories of pulp samples investigated in this study, as illustrated in Fig. 2.

**Table 1:** Properties of HC-TMP

Furnish	SPF
Net LC SRE (kWh/t)	0
Freeness (mL, CSF)	645
Mean fibre length (mm, L_W_)	2.04
Tensile index (Nm/g)	13.3
Bulk (cm^3^/g)	4.94
Tear Index (mNm^2^/g)	6.70

#### Baseline LC refining of high freeness TMP

The remaining pulp dispersed in the LC flow loop was LC refined with a coarse set of plates with 2.74 km/rev bar edge length (BEL), at an initial SRE of about 55 kWh/t per pass. Five samples were collected over 0–493 kWh/t SRE range at about 100 kWh/t SRE intervals and categorized as the first type of pulp: LCR-TMP.

#### Production of LC refined microfibres

Once the LCR-TMP samples were collected, the remaining TMP refined with 2.74 km/rev BEL and up to 493 kWh/t SRE was further refined to very high SRE targets using a finer set of plates with 12.90 km/rev BEL, for the production of highly refined TMP microfibre (TMPMF). The average refining energy and intensity for this stage of trial were about 49 kWh/t per pass and 0.05 J/m respectively. Microfibres collected at 1567 kWh/t and 2242 kWh/t SRE were later mixed with HC-TMP in different quantities to prepare the second and third categories of pulp, denoted by HC + TMPMF (1567) and HC + TMPMF (2242) respectively.

Similar to TMPMF production, NBSK pulp with a starting freeness of about 680 mL was LC refined in recirculation mode, initially with the 2.74 BEL plates, and later with the 12.90 BEL plates. KMF was collected at 2430 kWh/t SRE and mixed with different ratios of HC-TMP to prepare the fourth category of samples denoted by HC + KMF (2430).

### Sample preparation

Four categories of pulp samples were tested and compared with one another in order to understand the reinforcement potential of high energy microfibres in mechanical pulp-based paper products. The first category of pulp, LCR-TMP, was obtained through multistage LC refining of primary HC-TMP to five different specific energy targets, as listed in the leftmost column in Table 2. Each of the other three categories of pulp were prepared by reinforcing HC-TMP with one of the three microfibre types produced through excessive LC refining. Within each category, multiple samples were prepared by mixing a certain microfibre type with HC-TMP in different weight ratios so that each sample corresponded to a particular SRE target of the LCR-TMP. Since SRE of the HC-TMP is 0 kWh/t, SRE for these composite samples were calculated by multiplying the SRE of the microfibre by the weight percentage of microfibre used in preparation of that particular sample.

**Table 2:** LCR-TMP specific energy targets and wt% of HC-TMP and microfibres used in the composite samples

Specific energy target (kWh/t)	LCR-TMP		HC + TMPMF (1567)		HC + TMPMF (2242)		HC + KMF (2430)	
	LCR-TMP	HC-TMP	TMPMF (1567)	HC-TMP	TMPMF (2242)	HC-TMP	KMF (2430)	HC-TMP
	(wt%)	(wt%)	(wt%)	(wt%)	(wt%)	(wt%)	(wt%)	(wt%)
0	0	100^a^	–	–	–	–	–	–
113	100	0	7	93	5	95	5	95
228	100	0	–	–	10	90	–	–
315	100	0	20	80	14	86	13	87
387	100	0	–	–	17	83	–	–
493	100	0	31	69	22	78	20	80

^a^HC-TMP sample (primary pulp)

To illustrate, to match the energy target of 113 kWh/t, for the second category of pulp—HC + TMPMF(1567), 7.2% TMP derived microfibre collected at 1567 kWh/t LC SRE was mixed with 92.8% HC-TMP at 0 kWh/t LC SRE: 0.072 × 1567 kWh/t + 0.928 × 0 kWh/t = 113 kWh/t. Table 2 summarizes the LCR-TMP SRE targets as well as the ratios of microfibres and HC-TMP to achieve the various energy targets within each category of pulp.

### Characterization and testing

60 g/m^2^ non-recirculated handsheets were made using a conventional lab handsheet maker without any retention additives. Pulp drainage was measured using a Canadian Standard Freeness (CSF) tester. Handsheet preparation and testing as well as freeness determination were performed in accordance with TAPPI standards with minor deviations. For both handsheet preparation and freeness test, sample suspensions were heated to 80 °C temperature prior to disintegration for latency removal and fibre dispersion. For all samples, tensile index reported in result section was calculated based on oven-dry grammage, which interprets at about 10% higher tensile compared to the air-dry values. Samples were subjected to controlled temperature and humidity (CTH) at 25 ± 2 °C and 50 ± 3% respectively, for drying and testing.

Fibre dimensions were determined using a Fibre Quality Analyzer (FQA) manufactured by OpTest Equipment Inc. (Hawkesbury, Ontario, Canada). For all samples tested, fibres were defined as 0.200 mm–10.000 mm in length and fines were defined to be within 0.070 mm–0.200 mm range. Length weighted values were reported for both fibre length and distribution. Scanning Electron Microscopy (SEM) was used to examine the surface of handsheets at the 315 kWh/t SRE target for all four categories of pulp. Handsheet specimens attached to aluminum pin stubs by double sided adhesive tapes were coated with platinum (coating thickness: 8 nm) in a Cressington High Resolution Sputter Coater. SEM images were taken with a Hitachi S-2600N scanning electron microscope in high vacuum, at 300 times magnification and a viewing angle of 45° with respect to the plane of the handsheet surface.

## Results and discussions

Mechanical, optical and physical properties of three categories of microfibre composites as well as the baseline LCR-TMP samples are presented below. It should be noted that net power was used in the calculations of SRE for all samples in the experiment; the actual power consumption in the production of microfibres using LC refiner and in conventional multistage LC refining will be higher as it will include large refiner operational power typical of LC systems. As a reminder, the SRE for the composite samples refers to the value obtained by multiplying SRE of the microfibre by weight percentage of the microfibre in each composite sample. For composite samples, increasing specific LC refining energy also represents an increasing dosage of microfibre mixed with high freeness HC-TMP.

### Tensile development

**Fig. 3:**
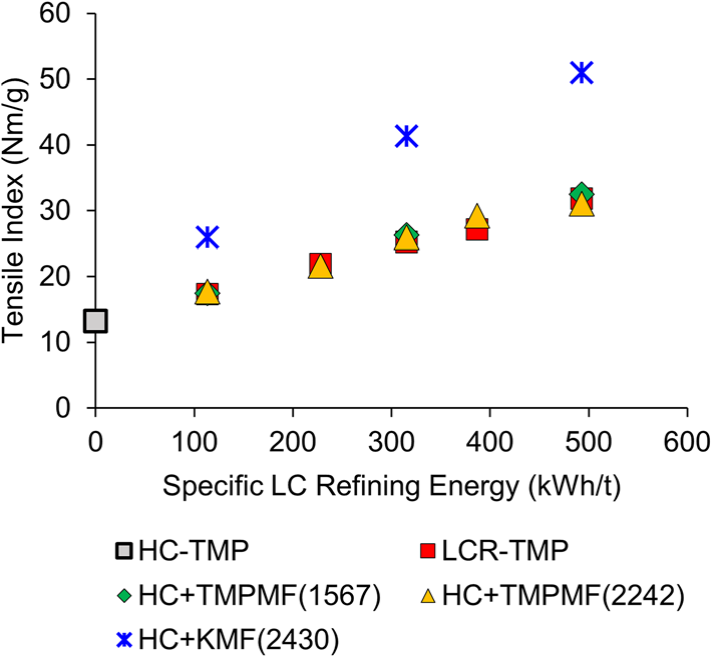
TMPMF addition to primary pulp resulted in tensile–SRE development similar to conventional LC refining while KMF addition shifted the curve significantly higher. A 20% addition of KMF at 2430 kWh/t SRE to primary HC-TMP resulted in 284% increase in tensile index

All four categories of pulp displayed an increase in tensile index with increased SRE, in Fig. 3. Primary HC-TMP reinforced with TMP-derived microfibres (  ,  ), exhibited tensile–SRE developments almost identical to conventional multistage LC refining (  ). This behavior is true for both 1567 kWh/t and 2242 kWh/t SRE microfibres. The addition of kraft-derived microfibre to HC-TMP (  ) yielded significantly higher tensile index at a given SRE. At equivalent energies, tensile gain from cellulose-rich KMF addition is 2–3 times greater than conventional LC refining and lignin-containing TMPMF reinforcement. The strength-enhancing potential of MFC produced from a variety of pulp furnishes is well documented in the literature (Brodin and Eriksen 2015; Djafari Petroudy et al. 2014; Eriksen et al. 2008; Meyer et al. 2018). The higher tensile index achieved by the addition of kraft MFC compared to TMP MFC has also been reported for kraft-based handsheets (Meyer et al. 2018). The current finding suggests the potential of LC-manufactured microfibres as an alternative to both multistage LC refining and expensive MFC reinforcement for mechanical pulp quality development.

### Pulp freeness

**Fig. 4:**
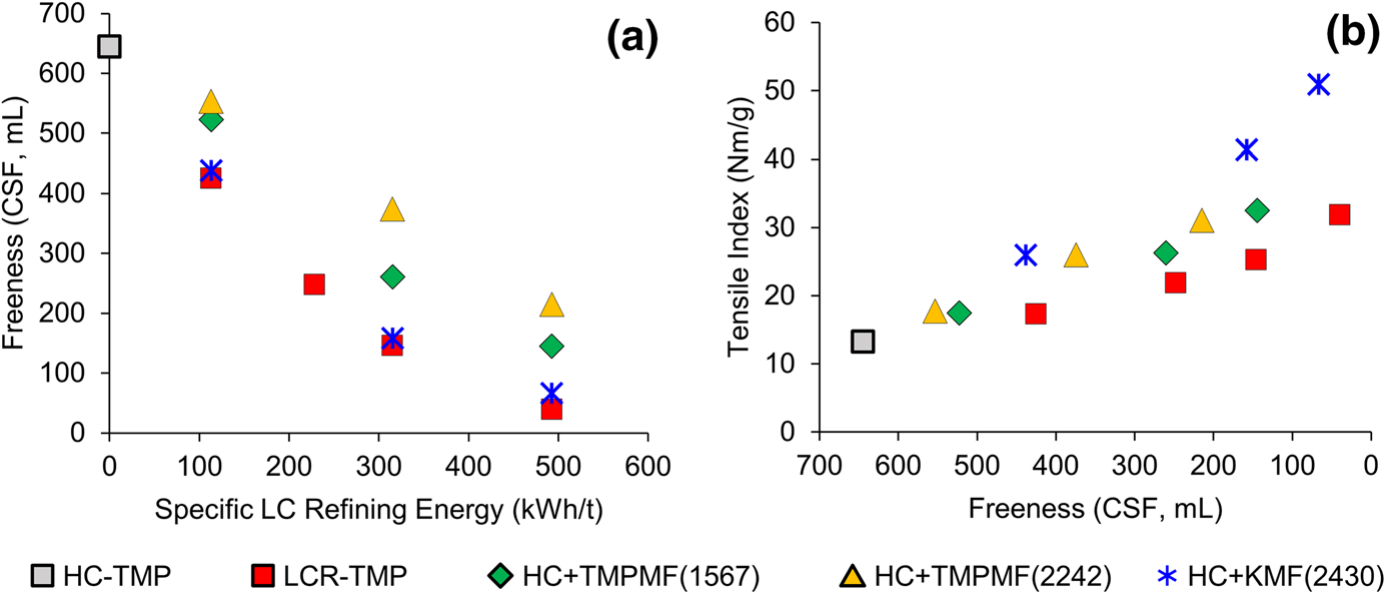
Freeness decreased with increasing SRE for all four categories of pulp (**a**); at a given freeness, tensile index is higher for microfibre composites compared to conventionally refined pulp (**b**)

In the composite samples. replacing long, intact fibres of primary HC-TMP with short, heavily refined microfibres caused a drop in pulp freeness, as observed in Fig. 4a. However, compared to LCR-TMP, composites made with the addition of TMPMF to HC-TMP (  ,  ) showed significant improvement in pulp drainability at a given specific energy. This is especially true for TMPMF at 2242 kWh/t SRE, as the higher SRE of this microfibre type required a smaller percentage of HC-TMP to be replaced to achieve certain SRE targets. In contrast, composites made with KMF (  ) showed very similar freeness drop to LCR-TMP (  ). At a given freeness, tensile strength was noticeably higher for all samples made with different microfibre types compared to conventional LC refined pulp, as demonstrated in Fig. 4b. The higher tensile development for composites made with KMF compensated for the higher freeness drop at a given specific energy.

### Bulk preservation

**Fig. 5:**
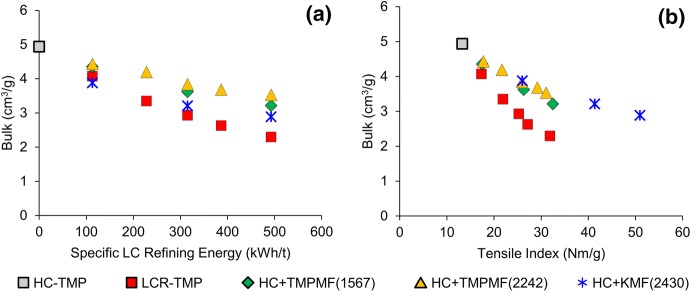
HC-TMP reinforced with different high energy microfibres preserved bulk better relative to conventional LC refined samples (**a**); at a given tensile, bulk is significantly higher for all microfibre composites compared to LCR-TMP (**b**)

Figure 5a shows reduction of bulk with increasing SRE for all four categories of samples. Increased fibre bonding through MFC and fine additions is known to reduce bulk of the reinforced handsheets (Brodin and Eriksen 2015; Eriksen et al. 2008; Meyer et al. 2018; Retulainen et al. 1993). At equivalent energies, microfibre additions to primary pulp yielded higher bulk compared to multistage LC refining. Increased fibre flexibility induced by refining, as well as higher specific surface due to fibre cutting, fibrillation and generation of fines during the refining process promote inter-fibre bonding (Gorski et al. 2012; Kerekes and Tam Doo 1985; Page 1989; Seth 2003) and lead to fibre network collapse and reduction of bulk (Forgacs 1963; Motamedian et al. 2019; Retulainen et al. 1993). Microfibre composites, on the other hand, were prepared by mixing long and intact fibres of primary pulp with a relatively small amount of highly developed fines and fibrils; this resulted in open construction handsheets with relatively higher bulk and improved mechanical strength. At a given SRE, the benefit of bulk retention is higher for composites made with TMPMF compared to KMF. Superior bonding ability of kraft fines (Retulainen et al. 1993), as well as its high fibre density, are likely to have contributed to the lower bulk of KMF composites.

The bulk–tensile relationship in Fig. 5b shows that at a given tensile, higher bulk was achieved for all three categories of microfibre composites (  ,  and  ) when compared to multistage LC refining (  ). The relatively lower bulk at a given SRE for KMF reinforced composites was compensated by the significantly higher tensile development for these samples. This overall improvement in bulk–tensile relationship through microfibre addition is remarkable since typical refining action and chemical treatments to increase the strength properties of TMP often result in large increase in sheet density, in other words, reduction of bulk (Chang et al. 2016a; Gorski et al. 2012). A few studies have shown that low-intensity LC refining and certain chemical treatments can improve bulk–tensile relationship (Chang et al. 2012; Miller et al. 2017); however, the benefit is often limited, especially compared to the microfibre addition to high freeness TMP in the current study.

### Improved tear strength

**Fig. 6:**
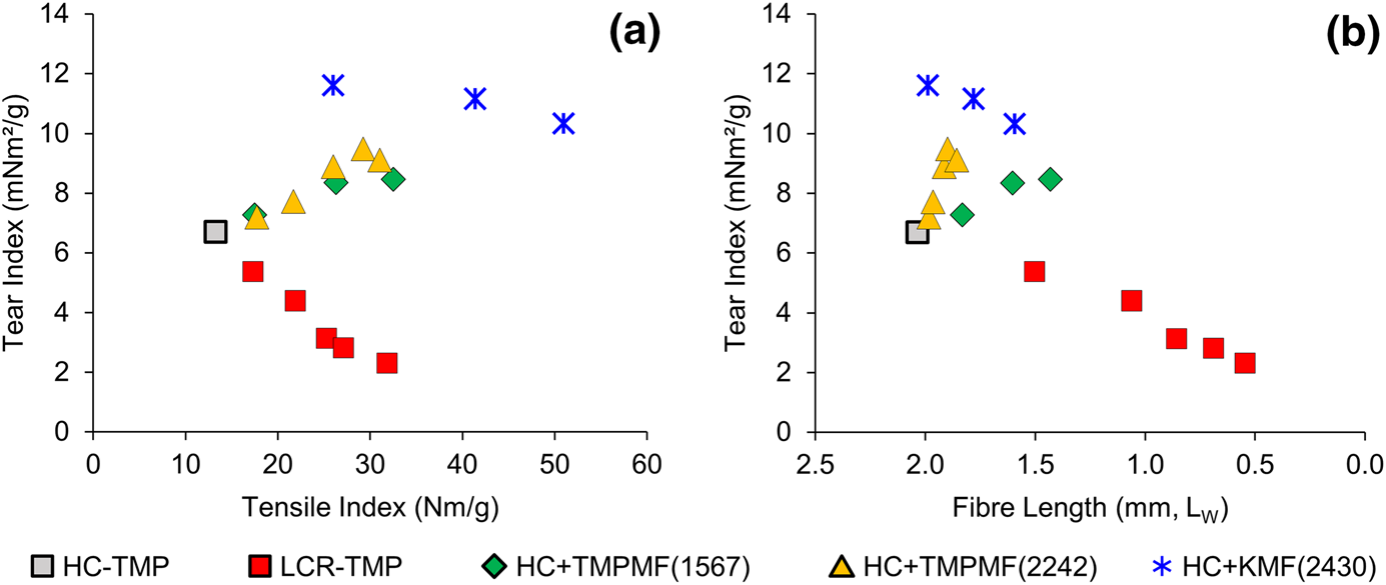
Tear index decreased with tensile for conventional refining but increased for microfibre reinforced composites (**a**); despite the gradual decrease in average fibre length, microfibre composites retained large quantities of long fibres of HC-TMP, which together with increased bond strength and improved network, contributed to the higher tear index of these samples (**b**)

Figure 6a demonstrates decrease in tear index with tensile increment for LC refined samples (  ). In contrast, microfibre reinforcement of primary pulp resulted in significant increase in tear index for all three categories of composite samples (  ,  and  ). The tear index of the microfibre composites started to decrease gradually at higher tensile strength, or to express differently, at higher microfibre additions, but remained significantly higher than the tear index of the primary pulp and the LCR-TMP. The highest tear index was achieved through KMF reinforcement of primary HC-TMP. These results are consistent with the study led by Meyer et al. (2018) which showed improved tear strength when MFC prepared from kraft fibres was used to reinforce TMP handsheets. The same study also reported increased tear index for kraft handsheets when different kraft and TMP grade MFCs were added, with kraft-based MFCs providing higher tear strengths. However, it should also be noted that a reduction in tear strength by MFC addition has also been reported in the literature (Djafari Petroudy et al. 2014; Eriksen et al. 2008).

One of the disadvantages of LC refining is fibre shortening and the resulting decrease in tear index with refining action, especially at higher intensities (Elahimehr et al. 2015; Hammar et al. 2010; Luukkonen et al. 2010; Seo et al. 2006). This was confirmed in the present study for LCR-TMP where tear index decreased with average length weighted fibre length of the samples, as demonstrated in Fig. 6b. In contrast, for composite samples of equivalent SREs, substituting long fibres of primary HC-TMP with heavily refined microfibres and fines resulted in overall reduction in fibre length but improved fibre-network and superior bonding. This in turn, resulted in higher tear resistance. For a given average fibre length, tear index was found to be the highest for KMF-reinforced composites and lowest for the LCR-TMP. This suggests the positive effect of bond strength and fibre network on tear index in addition to fibre length.

### Tensile energy absorption

**Fig. 7:**
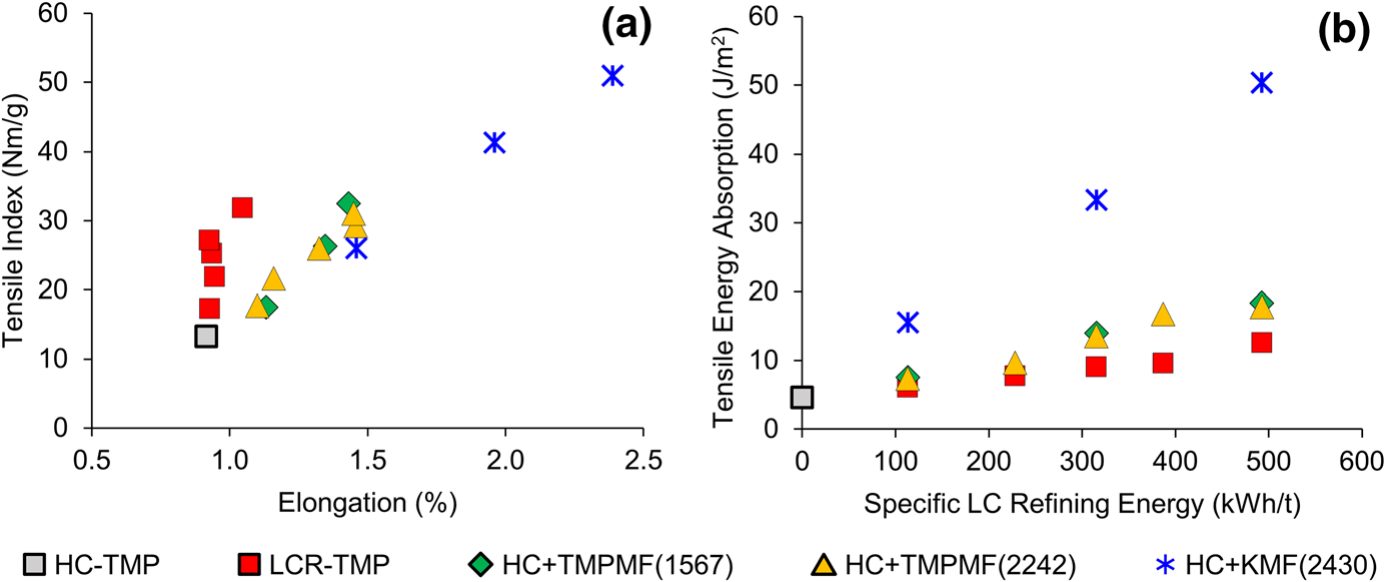
LCR-TMP and microfibre reinforced composites exhibited very different tensile–elongation behavior (**a**); tensile energy absorption (TEA) is significantly higher for KMF composites—the benefit of TEA from KMF reinforcement is about 6 times higher than LCR-TMP at 315 kWh/t SRE (**b**)

Additions of microfibre to primary HC-TMP resulted in higher elongation for the 60 grammage handsheets prior to rupture. Figure 7a shows an increase in elongation percentage with tensile index for all three categories of composite samples. LCR-TMP on the other hand, showed no significant change in elongation with tensile increment. Similar tensile development with specific energy (as seen earlier) but very different elongation behaviour of LCR-TMP and TMPMF composites suggests that tensile and elongation in TMPMF composites work very differently from LCR-TMP. Higher collapsibility of conventionally refined TMP resulted in very tightly formed handsheets where tensile index is likely to be a product of specific surface area and bonding potential of the constituent fibres and fines. In composite samples where long stiff fibres are glued together by highly developed fines and fibrils, part of the tensile strength possibly represents the ability of fibres to delocalize and distribute the stress over a large number of fibres and bonds. As a result, microfibre composites are better capable of withstanding rupture than LCR-TMP. In Fig. 7b, higher tensile energy absorption (TEA) confirms that microfibre composites are indeed more capable of enduring stress. Tensile index and elongation were both found to be remarkably higher for composites made with kraft microfibre.

### Light scattering

**Fig. 8:**
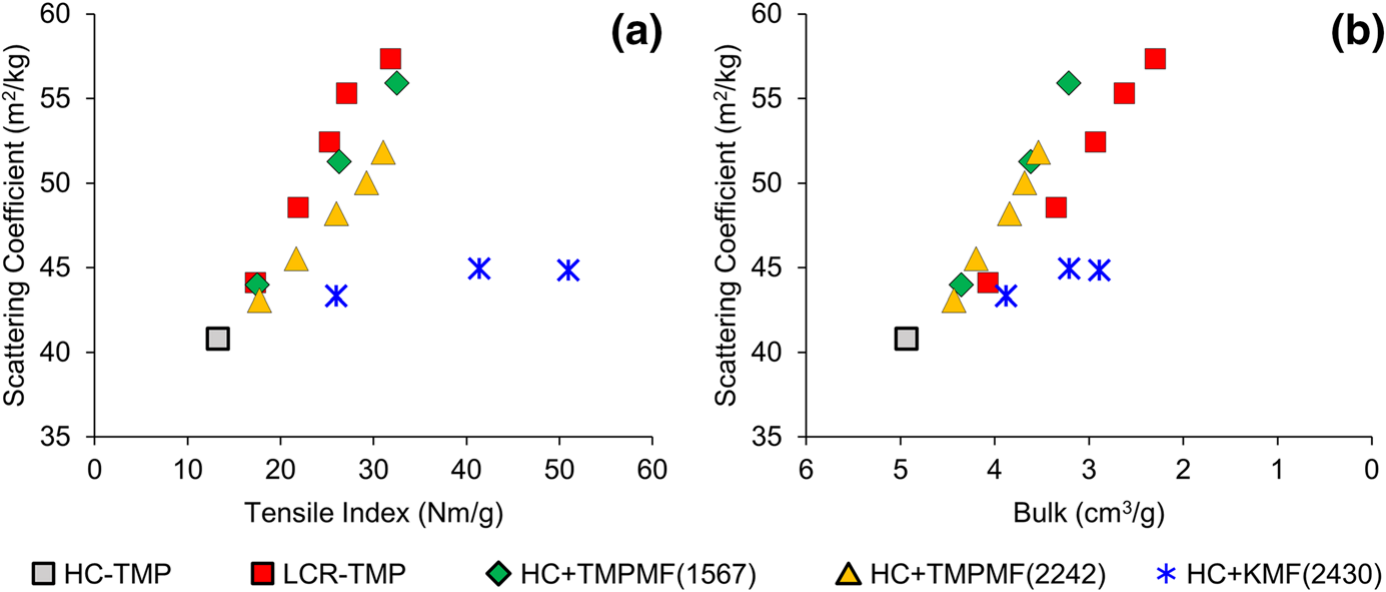
At a given tensile, higher light scattering was achieved for LCR-TMP samples compared to microfibre composites (**a**); at a given bulk, higher light scattering was gained through TMPMF addition to primary pulp as opposed to conventional refining (**b**)

Previous studies have shown that the addition of mechanical pulp fines and microfibrils to TMP furnish increased both tensile strength and light scattering (Brodin and Eriksen 2015; Retulainen et al. 1993), whereas the addition of kraft fines and MFC increased tensile but compromised light scattering coefficient (Eriksen et al. 2008; Meyer et al. 2018; Retulainen et al. 1993). In the present study, additions of both kraft and mechanical microfibre types improved tensile index and light scattering of high freeness HC-TMP, see Fig. 8a. Although the addition of KMF did not impair the light scattering of primary TMP in the limit of this study, scattering coefficient was substantially lower for KMF-reinforced composites (  ) than the other three categories of pulp. The highest scattering at a given tensile was achieved through the conventional LC refining of the high freeness primary pulp. However, at a given bulk, scattering coefficients were higher for TMPMF reinforced composites (  ,  ) than for conventionally refined LCR-TMP (  ), see Fig. 8b.

### Fibre length distribution

**Fig. 9:**
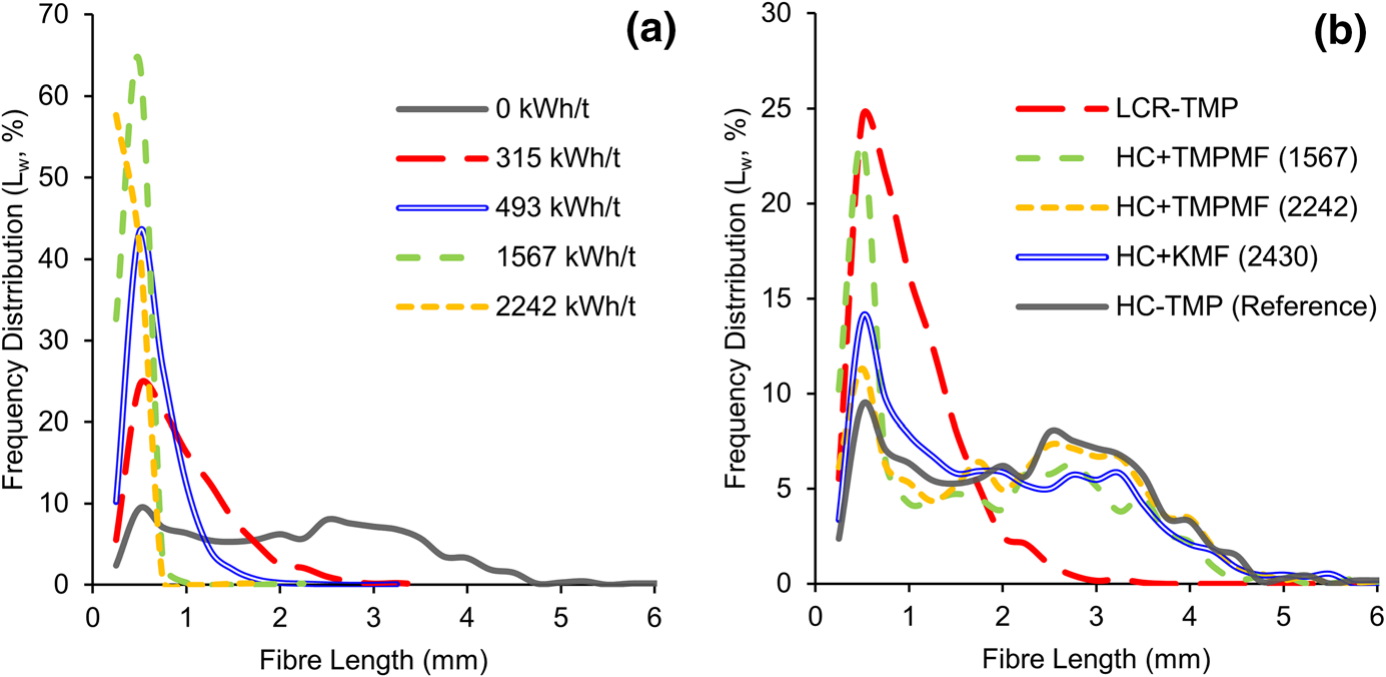
The fibre length distribution of primary TMP shifts gradually to the left with increased refining (**a**); a comparison of length distribution among the four categories of pulp at 315 kWh/t SRE and primary HC-TMP at 0 kWh/t SRE shows that the microfibre-reinforced samples resemble primary pulp more closely than the LCR-TMP samples (**b**)

Frequency distributions of fibres measured over 0.200–10.000 mm range were plotted against fibre length to show relative distributions of fibres in different pulp samples. Figure 9a demonstrates how length distribution of high freeness primary pulp evolved over 0–2242 kWh/t SRE range as energy was applied through multiple passes inside a pilot-scale disc refiner. With increased refining, long fibres were being cut and short fibres, in addition to fines and fibrils were generated; this in turn, shifted the distribution curve increasingly to the left of the plot. In case of primary pulp at 0 kWh/t SRE, about 50% of the fibres measured were between 2 and 4 mm length, and only about 12% of the fibres were in 0.20–0.50 mm range. In contrast, with heavy refining, at 2242 kWh/t SRE about 99% of the fibres measured fell between 0.20 and 0.50 mm length.

Figure 9b compares length distributions of four categories of pulp samples at 315 kWh/t SRE target, with that of primary HC-TMP at 0 kWh/t SRE as the reference pulp. In cases of microfibre composites where a small amount of long-fibred primary pulp was replaced with highly developed microfibres and fines, the distribution curve revealed the presence of large fractions of long fibres for these samples. At 315 SRE target, about 55–70% of the fibres measured in the different composite samples were greater or equal to 1 mm length. These long fibre fractions may have contributed to the improved tear strength and bulk discussed earlier. In contrast, LCR-TMP had the lowest fraction of long fibre—with about 30% of the fibres being greater or equal to 1 mm in length—and had the lowest bulk and tear among the four categories of samples.

Furthermore, composites made with the lowest SRE microfibres (1567 kWh/t) had the greater percentage of microfibres added to primary pulp—at 315 kWh/t SRE target, about 20% of TMPMF (1567) was mixed with 80% HC-TMP. This high microfibre content may be the reason for relatively higher light scattering for this category of pulp observed in the previous section.

### SEM characterization

**Fig. 10:**
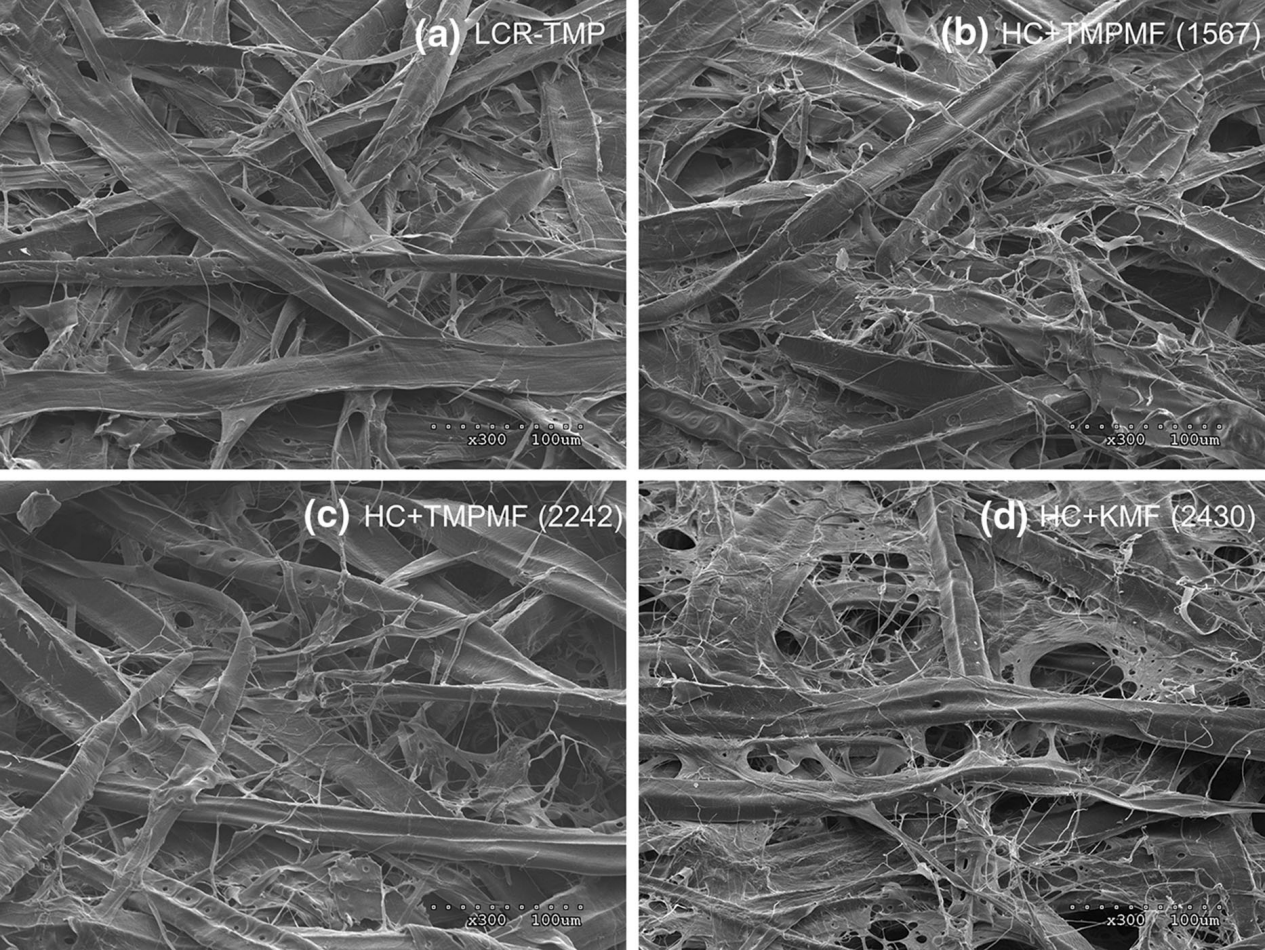
SEM images of handsheet surfaces of four categories of pulp, all at 315 kWh/t specific energy target: collapsed fibres created low-bulk handsheet for conventionally refined pulp (**a**), under-developed fibrils on the surface of handsheets prepared with TMPMF at 1567 SRE (**b**), long fibres of primary pulp covered in ribbon-type fibrils of TMPMF collected at 2242 SRE (**c**), hair-like fibrils of cellulose-based KMF traps long TMP fibres through web-like formations, in KMF reinforced composite (**d**)

Scanning electron microscopy (SEM) was used to investigate how TMP and kraft-derived microfibres interact with long fibres of primary TMP on the surface of 60 grammage handsheets. Images of these microfibre reinforced handsheets were also compared with the image of conventional LC refined handsheet to appreciate the difference in pulp property development among the different categories of pulp. All images presented in Fig. 10 represent surfaces of handsheets prepared with a 315 kWh/t SRE targets.

The handsheet surface of LCR-TMP in Fig. 10a shows conformed fibres with partial delamination characterized by the ribbon and hair-like fibrils mostly attached to the fibre surface. Increased fibre flexibility and delamination caused by the refining process increase the area of contact and mechanical interlocking between fibres. This promotes fibre collapse and the formation of rigid fibre networks, creating stronger but denser sheets with low elongation property (Fernando et al. 2013; Kang and Paulapuro 2006).

The surface of handsheets made with the addition of different microfibre types revealed ribbon and hair-like fibrils interacting with the fibres of primary pulp, in Fig. 10b–d. Small area of contact between stiff fibres of the high freeness pulp typically leads to weaker bonding and lower strength properties (Fernando et al. 2013; Forgacs 1963). The application of high specific energies through excessive LC refining breaks down fibre constituents and liberate a large number of fines and fibrils during microfibre production. The high specific surface of the hair-like and ribbon-type fines and fibrils together with their ability to access inter-fibre voids (Retulainen et al. 1993) allows for additional bonding between the long and stiff fibres of primary pulp in microfibre reinforced composite samples.

Additionally, the fibrils in the microfibre composites form web-like structures, in (b)–(d), and trap long fibres of the primary pulp, creating a tighter but open pore structure. This is especially true for microfibre prepared from kraft furnish—the thin hair-like fibrils of KMF (d), are long enough to drape over multiple fibres and overlap with other fibrils and fibre fractions—thereby expanding the fibre network. These KMF-reinforced handsheets can withstand higher stress by delocalizing and distributing stress over a large number of fibres and bonds, as discussed earlier. This, together with the superior bonding ability of kraft fines (Retulainen et al. 1993) results in significantly higher tear, tensile and TEA observed for the composite samples made from KMF additions to primary HC-TMP observed in this study.

The surfaces of handsheets made with the addition of TMP-based microfibres to primary pulp also revealed fibre-fibril interactions, in (b) and (c); however, these microfibres contained large number of ribbon-type fibrils, compared to KMF. The ribbon-type fibrils are characteristics of secondary S_2_ layers of fibre cell walls (Fernando et al. 2013; Forgacs 1963; Hafrén et al. 2014). The fibrils in mechanical pulp-microfibres appear to be more localized, covering relatively smaller number of intact fibres of primary TMP. This is reflected in the lower tensile and TEA of the TMPMF composites compared to the KMF counterpart. Additionally, fines and fibrils on the surface of handsheet made with the addition of TMPMF at 1567 SRE appear to be underdeveloped and not well-dispersed, compared to the surface of handsheet made with TMPMF at 2242 SRE. This may be the reason for the similar tensile development of these two categories of pulp, despite the very different microfibre addition rates at equivalent energies.

## Conclusion

The reinforcement potential of LC refined ligno-cellulose microfibres in mechanical pulp-based paper was investigated by observing pulp property development of high freeness primary TMP through different microfibre additions and by comparing it with conventional multistage LC refining of the same high freeness pulp. The major findings of this study were:

- Both mechanical and kraft microfibre types produced in a pilot-scale LC refiner improved mechanical properties of TMP-based handsheets.
- The addition of TMP microfibre to primary pulp resulted in tensile development almost identical to that of the conventionally refined LCR-TMP at equivalent specific energies. Additionally, TMP microfibre additions increased tear strength, preserved bulk and exhibited improved freeness when compared with conventional LC refined samples. Light scattering coefficient for these microfibre composites were lower than LCR-TMP at a given tensile but higher at a given bulk.
- Kraft microfibre additions to primary pulp produced composites with the highest tensile and tear strength in the course of this study. At a given tensile index, bulk and freeness were notably higher for KMF composites than conventional LC refined samples. The addition of KMF to primary pulp resulted in the lowest improvement in light scattering among the different categories of pulp tested.

This study explores the development and application of a unique mechanical pulp-based microfibre reinforcement and proposes an alternative to second stage LC refining of mechanical pulp for pulp property development. Excessive fibre cutting, bulk reduction and drop in pulp freeness brought about by conventional LC refining can be moderated by limiting the extent of secondary refining and strengthening mechanical pulp with high energy microfibres. Long-fibred chemical pulp has often been used as reinforcement for various mechanical pulp furnishes. However, chemical pulp is considerably more expensive than mechanical pulp. Commercially manufactured MFC is also expensive due to its high electrical and transportation cost. The use of LC refined TMP microfibres as reinforcement will allow TMP mills to take advantage of their existing resources for in situ production of microfibres to achieve high quality pulp. Similar tensile gain observed at a given energy target with multistage LC refining and TMP microfibre reinforcement suggests that the benefit of higher bulk, tear and freeness can be obtained without any additional energy consumption. The addition of highly refined kraft can further improve the pulp properties (with the exception of light scattering) and remains a feasible option if such improvement is desired.
